# Molecular Aspects of Inflammation and Lipid Metabolism in Health and Disease: The Role of the Mitochondria

**DOI:** 10.3390/ijms25126299

**Published:** 2024-06-07

**Authors:** Vasily N. Sukhorukov, Alexander N. Orekhov

**Affiliations:** 1 Petrovsky National Research Centre of Surgery, Moscow 117418, Russia; alexandernikolaevichorekhov@gmail.com; 2 Institute for Atherosclerosis Research, Osennyaya 4-1-207, Moscow 121609, Russia

Inflammation and lipid metabolism are two deeply interconnected and reciprocally regulated major physiological processes [1,2]. As such, both of these processes are fundamental for the onset and progression of many pathologies. The study of the interaction between the two is crucial for the identification of new therapeutic targets for patients with metabolic disorders, cardiovascular diseases, and chronic inflammatory diseases. It is important to note that the mitochondria, given their implication in inflammation and the release of danger-associated molecular patterns (DAMPs) as well as lipid metabolism fatty acid oxidation, triglyceride isolation, lipid synthesis, and lipid droplet interaction, can interconnect these two processes [3]. Moreover, it is known that the mitochondria are the main regulators of cellular metabolism and apoptosis [4]. In addition, damaged or malfunctioning mitochondria are increasingly recognized as the main cause of improper functioning of not only inflammation but also lipid metabolism [5,6].

The interaction between inflammation and lipid metabolism involves a number of sophisticated molecular mechanisms. First, major pro-inflammatory cytokines, such as TNF-α and IL-6, play an important role in the regulation of lipid metabolism. TNF-α can weaken the action of lipoprotein lipase and exacerbate the process of fat hydrolysis in adipocytes, thus increasing the number of free fatty acids in the bloodstream [7]. The pro-inflammatory cytokine IL-6 increases hepatic triglyceride production and alters lipoprotein function [8]. On the other hand, nuclear receptors such as PPARs are crucial for the regulation of lipid metabolism, but inflammation suppresses them and, as a result, disrupts lipid homeostasis [9]. The transcription factor NF-κB, activated by inflammatory signals, downregulates lipid metabolism genes while promoting pro-inflammatory gene expression [10]. In addition, eicosanoids, lipid mediators derived from arachidonic acid, can influence adipocyte function and insulin sensitivity, linking inflammation with lipid metabolism [11]. Adipokines such as leptin and adiponectin also mediate this interaction; inflammation elevates leptin levels, altering lipid metabolism and reducing insulin sensitivity while decreasing adiponectin levels, further dysregulating lipid metabolism [12]. The NLRP3 inflammasome, activated by metabolic stress, produces IL-1β and IL-18, exacerbating inflammation and disrupting lipid metabolism. Inflammation-induced insulin resistance impairs hepatic glucose suppression and lipid uptake, leading to an increase in circulating triglycerides and FFAs [13]. Toll-like receptors, particularly TLR4, activated by saturated fatty acids and lipopolysaccharides, initiate inflammatory pathways that interfere with insulin signaling and lipid metabolism. This complex interplay creates a feedback loop in which inflammation disrupts lipid metabolism and dysregulated lipid metabolism in turn exacerbates inflammation, driving metabolic diseases such as atherosclerosis, obesity, and type 2 diabetes [14].

Mitochondrial dysfunction triggered by inflammatory signals can impair fatty acid oxidation, which, in turn, can contribute to lipid accumulation and lipotoxicity [15]. Additionally, the presence of dysfunctional mitochondria can cause low-grade chronic inflammation [16,17]. The mitochondria are also the main signal transducers of the cell in response to nutrients, oxygen, and the cell cycle [18]. Defective mitophagy, an intracellular degradation of excessive or damaged mitochondria by means of autophagy, is one reason for the accumulation of dysfunctional mitochondria in a cell [19]. Therefore, their dysfunction is deeply involved in the onset and progression of many diseases. Mitochondrial dysfunctions often result from inherited or acquired defects in the mitochondrial genome, leading to defective fatty acid oxidation [15]. The accumulation of lipid intermediates typically triggers an inflammatory response through NF-κB and inflammasome activation. Damaged mitochondria can play a role in inflammasome activation by releasing DAMPs such as ROS, mtDNA, succinate, N-formyl peptides, cardiolipin, and ATP [20]. These DAMPs can trigger pro-inflammatory signaling pathways, such as the activation of the NLRP3 inflammasome and the release of pro-inflammatory cytokines like IL-1β and IL-18 [21]. Impaired mitochondrial function can disrupt cellular metabolism and bioenergetics, further enhancing the inflammatory response. Hence, constant exposure to inflammatory cytokines due to sustained mitochondria dysfunction is usually accompanied by chronic inflammation, serving as the main pillar of innumerable chronic inflammatory diseases, such as metabolic, neurodegenerative, and cardiovascular diseases [17].

Mitochondrial mutations can be the reason for dysfunctional mitochondria and/or defective mitophagy. Our recent study examined the impact of the m.15059G>A nonsense mitochondrial mutation on cellular functions relevant to atherosclerosis, including lipid accumulation, pro-inflammatory responses, and the process of mitophagy. Using the CRISPR/Cas9 system, adapted for mtDNA editing, we targeted the mutation and deleted it from the MT-CYB gene in human monocytic cell lines (Figure 1). The findings revealed that cells with the m.15059G>A mutation exhibited compromised mitophagy, reduced immune tolerance, and altered intracellular lipid metabolism due to increased FASN expression in monocytes and macrophages. This suggests the mutation may play a role in the development of atherosclerosis by interfering with lipid handling and immune responses within the cells [22].

The processes of inflammation and lipid metabolism play a major role in the development of a wide range of diseases, and the mitochondria are crucial to both. Dysfunctional mitochondria disturb both inflammation and lipid metabolic pathways, resulting in excessive lipid accumulation and increased inflammation. The fact that the m.15059G>A mutation is associated with cellular functions related to atherosclerosis implies a noticeable role of the mitochondria in a range of processes involving lipid metabolism and inflammation. Therefore, it can be assumed that new therapeutic approaches for managing metabolic and inflammatory diseases may evolve from the exploration of the links between the mitochondria and these processes.

## Figures and Tables

**Figure 1:**
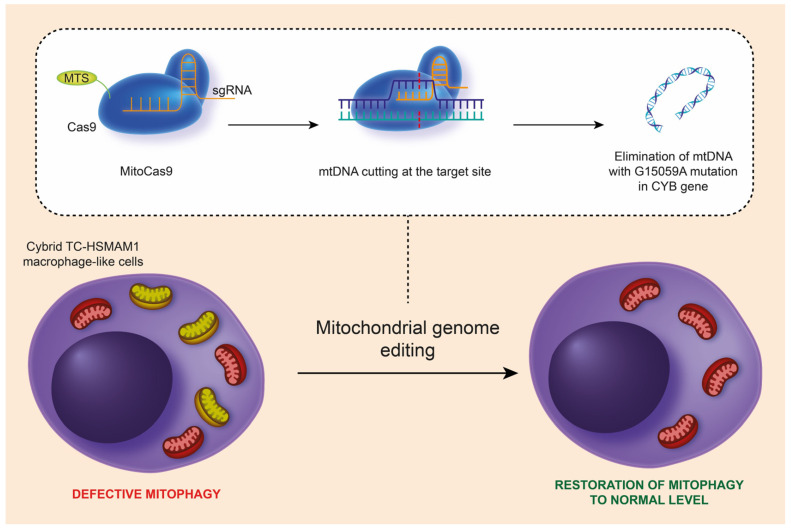
Schematic representation of the removal of the m.15059G>A mutation from mtDNA in hybrid TC-HSMAM1 macrophage-like cells using the CRISPR/Cas9 method, leading to the restoration of normal mitophagy levels in these cells. CRISPR/Cas9, clustered regularly interspaced short palindromic repeats; mtDNA, mitochondrial DNA; MTS, mitochondrial targeting sequence; sgRNA, single guide RNA; TC-HSMAM1, Thp1 Cybrid-High Sum Mutation Antiatherogenic Mutation 1. (This figure was reprinted with permission from [23], 2023, XHP Publishing).
